# A Central Hospitals Board for London

**Published:** 1893-01-28

**Authors:** 


### A CENTRAL HOSPITALS BOARD FOR LONDON.

[*From the Times of 25th inst.*]

THE conference on the recommendations of the Lords' Committee on Metropolitan Hospitals, held in July last at Spencer House, St. James's, reassembled yesterday evening at the offices of the London School Board, on the Victoria Embankment. Lord SANDHURST presided, and those present included Lord Cork, Mr. J. R. Diggle (Chairman of the London School Board), Mr. H. C. Burdett, Sir Douglas Galton, Mr. F. E. Villiers, Mr. J. B. Martin, the Rev. Dr. Troutbeck, Mr. J. P. Beckwith, Mr. E. Murray Ind, Mr. Timothy Holmes, Mr. R. P. D. Acland, Mr. H. Lucas, Mr. J. H. Hale, Dr. Sturgess, Mr. J. P. Wainwright, Dr. H. H. Tooth, Mr. H. M. Macpherson, and Mr. P. Michelli and Mr. C. L. Todd, the two honorary secretaries.

The report of the Special Committee appointed at the conference "to take into consideration the conclusions arrived at by the Lords' Committee on Metropolitan Medical Relief" was presented and read by Mr. MICHELLI. The committee stated that they considered the salient point was that of the formation of a central body of some description, and they appointed a sub-committee to deal with this portion of the subject. The sub-committee consisted of Lord Sandhurst, Sir Andrew Clark, Mr. Diggle, and Mr. H. C. Burdett. The sub-committee reported that they considered that it was practicable to form a central hospitals board in the spirit of the recommendations of the Lords' Committee. As to the constitution of such a board, it was suggested that the basis of the representation upon the board should be the committee of distribution of the Hospital Sunday and Saturday Funds. The latter committee, it was stated, numbered 14 members, and it was suggested that the Hospital Sunday Fund should be asked to add four members of their council to the 10 members of their distribution committee. It was further suggested that the representatives of the hospitals and dispensaries should be equal in number to those of the two funds combined—28. The committee recommended: (1) That each of the 12 endowed and general hospitals with medical schools should nominate one representative each; (2) that any other hospital not included in section 1, which had had a daily average of 150 beds occupied during the previous year, should nominate one representative each. At present, it was stated, five hospitals fell within this category. The remaining representatives should be elected by the medical charities participating in grants from the Hospital Sunday and Hospital Saturday Funds, upon the following basis—viz., each institution to be entitled to one vote for every £100, or part thereof, granted by the distribution committees of the Hospital Sunday and Saturday Funds. The sub-committee further suggested that each of the following eight bodies should be entitled to send one representative—viz., Royal College of Physicians, Royal College of Surgeons, the General Medical Council, Society of Apothecaries, General Practitioners, University of London, London County Council, and the Metropolitan Asylums Board. The board would therefore be composed of 64 members. The sub-committee, in considering what the functions of the central board should be, pointed out that no attempt would be made to administer the affairs of any hospital. It would also leave the Hospital Sunday Fund and the Hospital Saturday Fund in the same position as they now occupied as regarded constitution, collection, and distribution of funds. But it would give to these funds the advantage of being brought into direct contact with the best medical ability and with the best representatives of the active workers connected with the several institutions and with public bodies, and would tend in this way to a more complete understanding of the whole of the conditions under which the work of voluntary hospitals was performed, and the best means of supplying defects and initiating reforms. If the scheme thus foreshadowed should come into active existence, it might ultimately provide a common centre from which both the Hospital Sunday Fund and the Hospital Saturday Fund might conduct their operations, and at which provision might be made for the collection, in an easily accessible form, of information connected with the working of the medical charities of London and elsewhere. The sub-committee suggested that the name of the proposed central body should be—the London Central Hospital Board, the London Association of Hospitals, or the London Association of Medical Charities.

The CHAIRMAN stated that the resolutions in favour of the scheme were carried unanimously. Mr. Shaw Stewart wrote objecting to the scheme. His principal objection was that the committee had no permission on the part of the authorities of the Hospital Sunday and Saturday Funds to claim their support, but it was enough for the committee to know that they had the good will of the chairman of the Hospital Sunday Fund and of the treasurer of the Saturday Fund. A stronger protest had been received from Sir Douglas Galton, while Mr. W. Bousfield took objection to the proposals on points of detail. The general idea which prompted the sub-committee was that they should found a common basis of co-operation for every description of medical charity, and of bringing into line with them and one another the Hospital Saturday and Sunday Funds. Another thing they bore in mind was that in starting this new machinery they must be extremely careful to do so with the minimum of irritation to those bodies which they wished to assist to organise themselves into a line as charities. Objection had been taken to the introduction of the County Council and the Metropolitan Asylums Board. Sir Douglas Galton raised the objection that the duties of the proposed body were left in far too wide and open a condition. The committee considered that point very long and carefully, and came to the conclusion that they would more easily invite the co-operation of the medical authorities if they did not lay down any such stringent rule as that they were to go into this and that hospital and to do this and that. The committee, however, did consider that, supposing such a body to be formed from such institutions as were shown in the report, it was only a foundation, and that as its objects grew in public esteem and confidence, it would be able to map out duties for itself. He did not see why well-ordered institutions should fear interference. Sir Douglas Galton had pointed out that, in his opinion, the Hospital Saturday and Sunday Funds had a great deal too much power on the proposed board. (Cheers.) But the two funds had absolute power already in the distribution of money. His lordship concluded by moving the adoption of the report.

Mr. H. C. BURDETT seconded the motion.

Sir DOUGLAS GALTON said he objected to the report on two grounds—first, to the constitution of the committee, and, secondly, to the appointment of an influential committee without a clear definition of the duties which it was to undertake. (Cheers.) His first objection was based on the following reasons: A representative board meant one on which the different bodies who sent delegates would possess a degree of influence corresponding to some uniform standard. He objected to the proposal that the two funds should supply 28 members, while the whole of the hospitals together should only name an equal number; and, further, he thought the inclusion of eight members to be nominated by other bodies, of which at least four were unconnected with hospitals, was not founded on any reasonable ground. Upon what ground were the representatives of the two funds to have such a preponderance as to swamp the representatives of the hospitals? (Cheers.) As to his second objection, he said that there should be some clear definition of what the duties were which this body was to be called upon to perform, and some limits as to the extent of their interference with voluntary hospitals. The vague and elastic description of duties given in the report would admit of any amount of meddling with the affairs of the individual hospitals. What they wanted was a really representative body to deal with the many questions that required action. He therefore moved: "That the report be referred back to the committee, with a request that they will propose a scheme having a representative basis more in accordance with the report of the Lords' Committee than the scheme which they have submitted, and to report on the duties and powers of the proposed board."

Mr. W. BOUSFIELD seconded the amendment.

Mr. J. R. DIGGLE said that if the amendment were carried it would be impossible to have any central body at all.

Mr. ACLAND (Treasurer of the Hospital Saturday Fund) contended that the proposed board was a thoroughly representative one. If the recommendations of the Lords' Committee were adopted, two classes of persons would not be represented—the persons who contributed to the hospitals and the patients. Those two important bodies were, he urged, thoroughly represented by the Hospital Saturday and Sunday Funds. There was one excellent reason why the duties of the proposed board had not been defined, and that was that it should define them after it was established, and after conference with the authorities of the hospitals themselves.

In the course of further discussion which ensued representatives of the London and the Westminster Hospitals disclaimed any jealousy on the part of the hospital authorities in regard to the representatives of the Saturday and Sunday Funds.

Mr. HENRY C. BURDETT suggested the adoption of the following resolution:—"That this conference, approving in principle of the suggestion for the formation of the Central Hospitals Board for London, refer it to the sub-committe to communicate with the several institutions concerned in order to ascertain their views and their willingness to assist in forming the proposed central board."

Mr. TIMOTHY HOLMES said that the only practical course was to adopt the amendment of Sir Douglas Galton.

After further discussion the CHAIRMAN briefly replied to the points raised, and put the amendment of Sir Douglas Galton first. This was carried by a vote of 18 to 3.

Mr. DIGGLE moved that words approving in principle of the suggestion of the formation of a Central Hospitals Board for London should be inserted in Sir Douglas Galton's motion.

Mr. A. LUCAS seconded the proposal, which, on a division, was lost by 13 to 7.

Mr. ACLAND said another question now arose—whether the sub-committee would accept the reference. If the conference would not commit itself to the principle of a central board, it seemed waste of time to ask the sub-committee to hold a great number of additional meetings. As a member of the sub-committee, he felt great difficulty in acting.

Mr. BOUSFIELD hoped the sub-committee would act loyally in the matter.

Mr. DIGGLE suggested that the sub-committee should be increased by the addition of the names of six gentlemen who were in favour of the amendment which had been carried.

The CHAIRMAN said the meeting was one of the most extraordinary gatherings he had ever attended, for it was perfectly manifest that it was not the intention of this committee to have a central body at all. ("No, no.") However, he loyally accepted the decision of the majority.

Mr. HOLMES protested, amidst cheers, against his Lordship's interpretation of the views and intentions of those who had voted against the report of the sub-committee.

Mr. BURDETT moved,—"That the conference be adjourned until a date to be hereafter fixed, and that the sub-committee be instructed to obtain the opinions of the governing bodies of the institutions concerned on their report, amended in any way that may be considered desirable."

Mr. T. HOLMES seconded the motion, which was lost by 12 to 9.

The proceedings then terminated, after having lasted over two hours, with a vote of thanks to the chairman.

